# Comparison of RefSeq protein-coding regions in human and vertebrate genomes

**DOI:** 10.1186/1471-2164-14-654

**Published:** 2013-09-25

**Authors:** Jessica H Fong, Terence D Murphy, Kim D Pruitt

**Affiliations:** 1 National Center for Biotechnology Information, U.S. National Library of Medicine, 8600 Rockville Pike, Bethesda, MD 20894, USA; 2 Current address: 6425 Penn Ave. Suite 700, Pittsburgh, PA 15206, USA

**Keywords:** Genome annotation, RefSeq proteins, Protein quality assessment, Splice orthologs

## Abstract

**Background:**

Advances in high-throughput sequencing technology have yielded a large number of publicly available vertebrate genomes, many of which are selected for inclusion in NCBI’s RefSeq project and subsequently processed by NCBI’s eukaryotic annotation pipeline. Genome annotation results are affected by differences in available support evidence and may be impacted by annotation pipeline software changes over time. The RefSeq project has not previously assessed annotation trends across organisms or over time. To address this deficiency, we have developed a comparative protocol which integrates analysis of annotated protein-coding regions across a data set of vertebrate orthologs in genomic sequence coordinates, protein sequences, and protein features.

**Results:**

We assessed an ortholog dataset that includes 34 annotated vertebrate RefSeq genomes including human. We confirm that RefSeq protein-coding gene annotations in mammals exhibit considerable similarity. Over 50% of the orthologous protein-coding genes in 20 organisms are supported at the level of splicing conservation with at least three selected reference genomes. Approximately 7,500 ortholog sets include at least half of the analyzed organisms, show highly similar sequence and conserved splicing, and may serve as a minimal set of mammalian “core proteins” for initial assessment of new mammalian genomes. Additionally, 80% of the proteins analyzed pass a suite of tests to detect proteins that lack splicing conservation and have unusual sequence or domain annotation. We use these tests to define an annotation quality metric that is based directly on the annotated proteins thus operates independently of other quality metrics such as availability of transcripts or assembly quality measures. Results are available on the RefSeq FTP site [http://ftp.ncbi.nlm.nih.gov/refseq/supplemental/ProtCore/SM1.txt].

**Conclusions:**

Our multi-factored analysis demonstrates a high level of consistency in RefSeq protein representation among vertebrates. We find that the majority of the RefSeq vertebrate proteins for which we have calculated orthology are good as measured by these metrics. The process flow described provides specific information on the scope and degree of conservation for the analyzed protein sequences and annotations and will be used to enrich the quality of RefSeq records by identifying targets for further improvement in the computational annotation pipeline, and by flagging specific genes for manual curation.

## Background

The large number of genomes that have been sequenced in recent years has been followed by an unprecedented amount of data at all levels of biological systems, from genomic assemblies to gene annotations and mRNA and protein sequences. Collections of high-quality biological data include curated genomic, transcript, and protein records in the National Center for Biotechnology Information’s (NCBI) Reference Sequences (RefSeq) database [1]; consistently annotated human and mouse protein-coding regions (CDS) in the Consensus Coding Sequence database (CCDS) [2]; and curated protein data in Swiss-Prot/UniProtKB [3]. Outside of the best-studied species such as human and mouse, much of the available annotation data is predicted using computational pipelines or high-throughput techniques, with or without supplemental manual curation, and may therefore include more frequent errors [4]. At the same time, the increasing quantity and expanding scope of biological data enables assessment of conservation, evolutionary histories, and functional importance using comparative genomics and proteomics. Comparative genomics is becoming indispensable and has recently been applied toward problems in gene annotation and evolution, such as distinguishing coding from non-coding genes [5], identifying functional elements [6], and modeling the evolution of vertebrate exons and introns [7].

With the exception of human and mouse, most vertebrate RefSeq transcripts and proteins were predicted using NCBI’s eukaryotic annotation pipeline component. Gnomon is the core computational tool which integrates analysis of transcript and protein alignments and *ab initio* data to generate a set of annotation models which are further filtered before being selected for final RefSeq genomic annotation [8]. Although this method tends to produce gene and protein annotations that match known ones, it also results in annotation differences that are supported by additional data available for one genome over another, or differences that are influenced by insufficient same-species transcript evidence, genome assembly issues, inexact exon definitions based on protein alignments, or limitations of the prediction method.

The Vertebrate RefSeq project has developed a conservative protocol for comparative analysis of proteins in order to assess computational annotation of RefSeq proteins and to supplement quality assurance measures. Our protocol identifies orthology at the level of annotated RefSeq vertebrate genomes. It leverages the sizable collection of genomic, transcript, and protein sequences in the RefSeq database to assess consistency and conservation of protein sequences, domain annotations, and annotated protein-coding sequence (CDS) regions on RefSeq genome sequences across sets of orthologs, while accommodating for diversity in splicing products across genes and wide differences in data quality across existing annotations. As a critical part of our study, we evaluate conservation at two orthogonal levels: protein sequence and gene structure; that is, protein-coding regions on the gene. Changes in amino acid sequence and the translated coding region and exons of the respective genes are driven by different molecular mechanisms (largely mutations vs. exon shuffling). Consequently, integrating analysis of sequence similarity and coding regions helps to detect and distinguish changes involving whole exons from localized mutations and indels. Conservation at the splice level has been used toward novel gene finding, particularly to detect homologs and to predict intron-exon structure [9,10]. Our work extends these previous studies by including many other vertebrates and by integrating evaluation of all splicing isoforms rather than a selected protein for each gene. We determine “splicing orthologs” [9] (namely, isoforms with the same pattern of protein-coding exons) in vertebrates by aligning protein-coding regions in protein sequence space.

In this report, we describe the application of our protocol towards three specific aims. First, we identify those proteins with sequences and splice patterns consistent with its orthologs, indicating correct annotation. When these proteins are present in a large number of species across some taxonomic scope (here, across mammals or vertebrates), we also designate them as “core” proteins that are expected to be consistently found in novel genomes and may have high functional importance over their conserved lineage [11,12]. Second, we search for proteins with inconsistent amino acid sequences compared to their orthologs in order to identify targets for improvements to the computational annotation pipeline and/or for curatorial review. We describe a suite of computational screens which assesses sequence lengths over whole protein and terminal regions, sequence similarity, domain composition, and closest neighbors in other species. Lastly, we explore the effectiveness of combining our measurements to infer the quality of predicted annotations and assemblies.

## Results and discussion

### Computational pipeline

**Figure 1:**
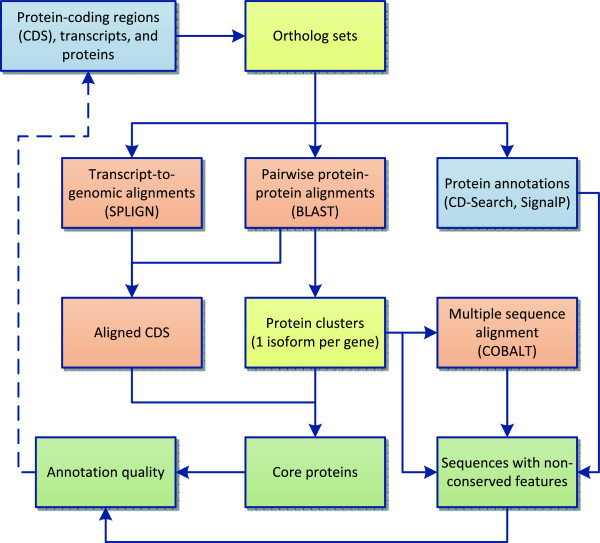
**Process flow diagram.** Input data is indicated by the blue background. Some data elements included in this analysis are pre-calculated and available as feature annotation on public sequence records, other elements are calculated as part of our analysis. Orange boxes indicate alignment processes, and other methods are indicated by yellow boxes. Analysis outputs are indicated at the bottom in green.

We have developed a process flow that utilizes the RefSeq sequence collection to explore questions of gene and protein conservation and annotation consistency in vertebrate RefSeq genomes (Figure 1). In brief, this protocol identifies sets of comparable proteins at different levels including orthologous genes as well as most similar proteins among multiple alternatively splicing products, and then it integrates sequence, CDS, and functional annotation (via conserved domains) to evaluate conservation from multiple viewpoints.

We take particular care to address challenges related to data quality, availability, and scalability in dynamic databases. First, requiring only sequence data allows the process to be applied to the widest number of proteins, transcripts, and genome annotations available in NCBI databases. In particular, it avoids using external data sources such as whole-genome alignments from the UCSC Genome Browser [13], which are less frequently updated. Cross-species alignments are efficiently calculated over protein space using BLAST software. Subsequently, transcript sequences and annotated CDS may be mapped onto their respective protein sequence alignments to determine splice conservation across orthologs. Second, our protocol minimizes re-calculations in the course of database updates and takes advantage of pre-computed domain assignments and protein-coding regions when available. Additionally, all proteins are benchmarked against a small set of reference species believed to have higher quality assembly and protein data. These species have been selected to be somewhat well distributed across sequenced vertebrates and are estimated to be of higher quality according to the number of ESTs, contig N50, scaffold N50, curated data, and by our own evaluation.

### Identification of orthologs

RefSeq calculates sets of orthologous genes from full proteomes by taking best hits to Swiss-Prot proteins as a set of potential homologs and then confirming orthology through local synteny, as described in Methods. Combining protein homology and genomic co-localization, two largely independent methods, provides an efficient, large-scale method for identifying true orthologs, although this approach may miss genes that have undergone greater divergence or are poorly represented or annotated in protein databases [14]. Our approach calculates orthologs during each genome annotation run to support rapid analysis of new protein datasets as additional vertebrate organisms are annotated. Compared to the HomoloGene algorithm [15,16], the method used here is 99.6-99.9% consistent, while finding orthologs for an additional 2-14% of genes (Table 1). Our method finds more than 15,000 orthologs for most mammalian genomes, but is less efficient for non-mammalian vertebrates owing to shorter syntenic blocks and the reliance on best hits to the Swiss-Prot database which has better representation for mammalian proteins.

**Table 1:** Comparison of RefSeq orthology vs. HomoloGene

**Organism**	**RefSeq orthologs**	**Avg. synteny support**^**1**^	**HG orthologs**	**Orthologs that agree with HG**	**Orthologs in conflict with HG**	**Percent that agree with HG**	**RefSeq orthologs not in HG**	**In HG, not in RefSeq orthologs**
Chimpanzee	17070	4.7	16037	15042	14	99.9%	1937	995
Mouse	16174	5.0	16514	15605	60	99.6%	407	909
Rhesus macaque	15981	4.4	15144	13668	39	99.7%	2146	1476
Cow	15910	4.7	15813	14781	38	99.7%	974	1032
Dog	15890	4.7	15904	15016	40	99.7%	721	888
Rat	15231	4.4	15200	13480	51	99.6%	1566	1720
Chicken	11201	4.1	12141	10618	30	99.7%	497	1523
Zebrafish	5799	2.1	10786	4851	11	99.8%	667	5935

Comparisons are made to HomoloGene (HG) clusters containing a single gene from human and the indicated species, using data from HG Release 67 (Dec 13, 2012).

^1^out of 6 flanking loci assayed for each orthologous gene.

**Figure 2:**
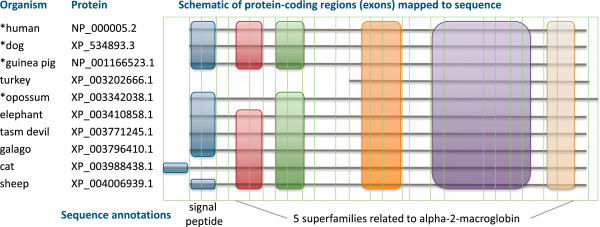
**Human *A2M *alpha-2-macroglobulin (GeneID 2) and its orthologs from 9 species.** Reference species are marked with *. Each of these genes encodes a single RefSeq protein. This schematic shows the sequence alignment. Protein-coding regions were mapped to sequence with vertical green lines showing splice boundaries (7 proteins have 36 coding exons; others are truncated or extended). In reality, splice boundaries will not all align perfectly. Annotations predicted from sequence include signal peptide and five distinct CDD superfamilies.

**Figure 3:**
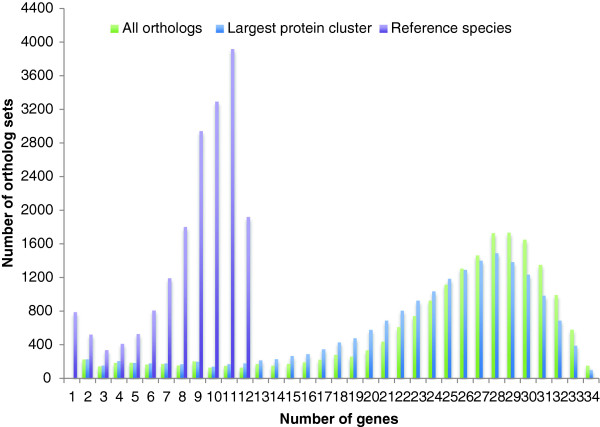
Histogram of the number of genes in each ortholog set for all species, reference species, and the number of genes in the largest protein cluster within each ortholog set.

We report results on the 18,481 ortholog sets (453,209 genes) current at the time of this analysis. The orthology dataset contains 568,459 RefSeq proteins of which 19% are in the pool available for manual curation (with NP accessions; of which half have been curated). Each ortholog set was assembled with a human gene as “anchor” for all other genes, plus one or more orthologs from the 33 other vertebrate species evaluated (Table 2). During a small time lag between the initial assembly of ortholog sets and subsequent protein analysis, 60 human anchoring genes were dropped from the analysis dataset due to ongoing RefSeq curation which updated the protein accession.version. The majority human-anchored ortholog sets represent a highly comprehensive set of protein-coding genes within the RefSeq collection, including 18,421 (or 94%) of all human protein-coding genes. Figure 2 illustrates the set of orthologs anchored around human gene alpha-2-macroglobulin showing how genomic protein-coding regions are mapped onto protein sequence, along with the conserved domains and signal peptide features predicted from sequence. The distribution of genes in ortholog sets is illustrated in Figure 3. The median number of species in ortholog sets is 27 and 92-96% of sets contain at least 10 and 6 species, respectively.

**Table 2:** Ortholog dataset used in current analysis

**Tax ID**	**Organism**	**Annotation release date**	**Genes in ortholog dataset**	**Annotated protein-coding genes**	**% Pipeline prediction**	**EST count**	**Assembly accession**	**Contig N50**	**Scaffold N50**
7955	*zebrafish	3/24/2011	5481	26329	48	1,481,937	GCF_000002035.4	1,073,451	1,551,602
8128	nile tilapia	9/30/2011	5898	22130	100	120,196	GCF_000188235.1	29,493	2,802,423
8364	western clawed frog	7/29/2010	9611	21989	62	1,271,375	GCF_000004195.1	17,038	1,567,461
9031	*chicken	12/16/2011	11170	16725	71	600,433	GCF_000002315.3	279,750	12,877,381
9103	turkey	3/25/2011	8981	12129	100	17,435	GCF_000146605.1	12,520	857,645
9258	platypus	9/3/2011	6917	16477	99	9,699	GCF_000002275.2	11,554	958,970
9305	tasmanian devil	7/16/2012	12456	19365	100	0	GCF_000189315.1	20,139	1,847,106
9483	white-tufted-ear marmoset	6/8/2012	15191	19408	100	2,605	GCF_000004665.1	29,293	5,167,444
9544	rhesus macaque	6/2/2010	15228	22541	97	58,412	GCF_000002255.3	25,707	6,094,595
9555	olive baboon	9/5/2012	15583	21785	98	145,582	GCF_000264685.1	40,262	528,927
9593	western gorilla	12/6/2012	16250	22059	100	0	GCF_000151905.1	11,661	913,458
9597	pygmy chimpanzee	7/25/2012	16519	20463	100	0	GCF_000258655.1	66,775	10,124,892
9598	chimpanzee	10/27/2012	16997	21396	96	17,130	GCF_000001515.5	50,679	8,925,874
9601	sumatran orangutan	7/18/2012	14981	22822	86	46,981	GCF_000001545.4	15,648	747,460
9606	*human	10/30/2012	18421	19527	2	8,699,560	GCF_000001405.22	38,508,932	44,983,201
9615	*dog	2/2/2011	15784	19163	93	382,638	GCF_000002285.3	267,478	45,876,610
9646	giant panda	7/30/2010	14466	17892	100	0	GCF_000004335.1	39,886	1,281,781
9685	domestic cat	11/7/2012	15864	18201	98	919	GCF_000181335.1	20,621	4,658,941
9785	*african savanna elephant	8/25/2011	14259	18389	100	0	GCF_000001905.1	69,023	46,401,353
9796	*horse	6/28/2011	14668	18002	96	37,199	GCF_000002305.2	112,381	46,749,900
9823	pig	10/11/2011	12283	21992	84	1,624,129	GCF_000003025.5	69,669	576,008
9913	*Bos taurus (bovine)	12/2/2011	16013	21157	39	1,559,494	GCF_000003055.4	96,955	6,380,747
9940	sheep	12/2/2012	15588	19097	96	338,483	GCF_000298735.1	40,376	100,079,507
9986	rabbit	4/23/2010	9032	16117	94	34,938	GCF_000003625.2	64,648	35,972,871
10029	chinese hamster	10/17/2011	13835	19702	99	0	GCF_000223135.1	39,361	1,147,233
10090	*house mouse	10/1/2012	16142	21780	6	4,853,5*8	GCF_000001635.21	32,273,079	52,589,046
10116	*norway rat	6/20/2012	15718	22719	29	1,103,577	GCF_000001895.4	59,469	2,178,346
10141	*domestic guinea pig	10/3/2011	14436	18029	98	19,975	GCF_000151735.1	80,583	27,942,054
13616	gray short-tailed opossum	5/31/2011	12942	17924	98	265	GCF_000002295.2	108,014	59,809,810
27679	*bolivian squirrel monkey	9/9/2012	16089	19331	100	0	GCF_000235385.1	38,823	18,744,880
28377	*green anole	3/30/2011	10041	15645	100	156,802	GCF_000090745.1	79,867	4,033,265
30611	small-eared galago	7/18/2012	15596	19454	100	0	GCF_000181295.1	27,100	13,852,661
31033	torafugu	11/6/2012	5560	18592	98	0	GCF_000180615.1	52,883	928,938
61853	northern white-cheeked gibbon	5/6/2011	15209	19556	100	0	GCF_000146795.1	35,148	22,692,035

The assembly name, assembly accession, contig N50, and scaffold N50 are reported from the NCBI Assembly resource. The % pipeline prediction column indicates the percent of annotated computationally predicted proteins (XP accession prefixes) out of the total annotated proteins (XP and NP accession prefixes). Reference species are flagged with * in the Organism column. EST count refers to the number of same-species ESTs that were available at the date of the annotation run; some annotation runs also used cross-species transcript data or 454 RNAseq data.

All proteins from each set of orthologous genes are partitioned into clusters containing at most one protein from each species so that each protein is grouped with the most similar proteins from other species according to sequence similarity and length. Limiting comparisons of proteins to those within the same cluster is intended to reduce spurious differences from comparing homologous proteins that are not the closest relative to one another. Figure 3 shows that the largest clusters are slightly smaller than the full ortholog sets; the median number of species in each largest cluster is 25, however again 92-96% of sets contain 10 and 6 species, respectively.

The 34 species reported here represent four taxonomy subgroups: primates (11 species), rodents (5 species), other mammals (11 species), and non-mammalian vertebrates (7 species). From these species, a subset of 12 species believed to have higher-quality data and representing all four subgroups were selected to use as reference genomes: human and Bolivian squirrel monkey (primates); mouse, rat, and guinea pig (rodents); dog, elephant, horse, and cow (mammals); and zebrafish, chicken, and anole (vertebrates). The reference species contain 205,670 proteins. For full assembly information for the organisms evaluated here, see Table 2. This table also describes the version of the assembly and annotation that is represented in the reported dataset and additional details on the protein-coding annotation results, statistical metrics that are commonly used to evaluate the quality of the assembly, and one measure (EST count) of the amount of same-species transcript data that was available for that annotation run.

The Conserved CDS database (CCDS) [2] contains human and mouse protein coding regions that are consistently annotated in NCBI and Ensembl genome browsers and provides a gold standard for coding region locations. Comparison of human genes in our orthology dataset and in CCDS shows that the intersection of the two dataset contains 92% of all human proteins in orthology sets and 86% of human proteins in CCDS. For mouse, the respective values are 93% and 80%. This sizable overlap confirms that nearly all human and mouse genes in the orthology dataset are likely to have valid CDS. The CCDS proteins that do not overlap with our ortholog dataset relates to our method of determining ortholog sets, which tends to omit paralogous gene clusters and large gene families with notable species expansion differences (e.g., olfactory receptors).

### Splicing conservation in vertebrates

#### Integrated sequence-splicing method for identifying highly conserved orthologs

To measure splice-level conservation in protein-coding regions, the splice boundaries of transcripts were obtained from genome annotations or mRNA-genome alignments calculated using the Splign program [17]. Coding regions and their splice boundaries are compared in protein space in order to use protein-protein alignments to determine corresponding genomic positions, similarly to [18]. We define two proteins as splicing orthologs if all protein-coding exons in the two proteins can be paired with 90% overlap in lengths of both exons. Our approach ensures that splicing orthologs exhibit sufficient sequence similarity at the level of every individual protein-coding exon and very similar CDS splice patterns, allowing more flexibility for insertions and deletions than sequence-independent methods such as Exalign [19]. Unlike methods that depend on pre-calculated whole-genome alignments to assess intra- or intergenic regions across species [13], our software can be applied to any valid sequence, in parallel to revision of existing sequence records and newly deposited proteins.

To compare the extent of splicing conservation according to our method and others, we gathered a subset of our dataset consisting of 15,511 protein clusters with human and mouse orthologs, as well as the 13,418 cow and 4617 zebrafish proteins in these clusters. Only one cluster is evaluated for each ortholog set (using the largest cluster containing both human and mouse proteins), and a single protein from each species. Our testing showed that 71% of mouse proteins, 68% of cow proteins, and 27% of zebrafish proteins are splicing orthologs of an annotated human RefSeq protein (see Tables 3 and 4). The former is comparable to the 64% fraction of splicing orthologs between human and mouse that was previously reported [9], among human-mouse transcripts with at least 4 protein-coding exons. We also consider whether exon splice junctions are aligned; this test yields slightly higher conservation rates, as may be expected, but only applies to proteins with multiple coding exons (87-90% of proteins from mouse, cow, and zebrafish have human ortholog with at least 3 coding exons). These results indicate that a method based on fraction overlap provides enough flexibility to detect splicing orthologs even in species as distant as human and zebrafish.

**Table 3:** Protein and conserved splicing attributes, numbers of proteins in evaluation dataset for splicing conservation satisfying different conditions

**Organism**	**Number of proteins**	**All coding exons aligned**	**1-to-1 mapping between exons**	**All exons have 90% overlap**	**All exons have length +/− 15**	**Proteins with > =3 coding exons**	**All splice junctions aligned**
Mouse	15511	14275	13638	11074	10422	13451	10639
Cow	13418	11581	10697	9073	8572	11680	8407
Zebrafish	4617	3385	2900	1264	1307	4155	1805

**Table 4:** Protein and conserved splicing attributes, specific differences accounting for absence of conserved splicing as we measure it

**Organism**	**Proteins without 90% overlap**	**Exon split/merge**	**Exon loss**	**Not all coding exons aligned**	**All exons 1-to-1 but lengths vary**
Mouse	4437	328	573	1236	2564
Cow	4345	699	679	1837	1624
Zebrafish	3353	522	381	1232	1636

Inspecting the human-mouse, human-cow, and human-zebrafish ortholog pairs that lack conserved splicing provides some insights into why some orthologous proteins are not splicing orthologs. First, 37-58% of ortholog pairs have all coding exons paired one-to-one yet at least one pair of exons differs by over 90% in length. An additional 28-42% of ortholog pairs have protein alignments that exclude at least one protein-coding exon. This may occur due to data quality issues in the genome assembly, lack of high quality transcript or protein evidence, or low sequence similarity. For example, lower sequence similarity in terminal regions may exclude those regions from the protein alignment and consequently the corresponding CDS. Also, certain mechanisms such as exonization, exon shuffling, or intronization are known to create novel coding exons or to merge or split exons. By searching for exons with no counterpart in the ortholog or split exons in one transcript mapped to a single exon in the other (including both single-exon and multi-exon genes), we find that 20-30% of non-splicing orthologs may have undergone these mechanisms. However, these differences may also be due to errors from our annotation pipeline or in the genome assembly at that gene location.

The above results do not change if we restrict the evaluation set to the 4247 clusters that contain one protein from each of the four species; in that case, a very slightly higher fraction of proteins show conserved CDS (data not shown). We also verify that splicing conservation and sequence conservation are complementary measures. Over pairs of orthologous proteins from human and each of the other species, and excluding protein pairs with perfectly conserved splicing which contribute a large number of tied scores, the Pearson correlation coefficient between sequence identity (number of identical residues over alignment length) and fraction of exons conserved (with 90% overlap) is a weak 20-42%.

#### Conserved CDS in vertebrates

**Figure 4:**
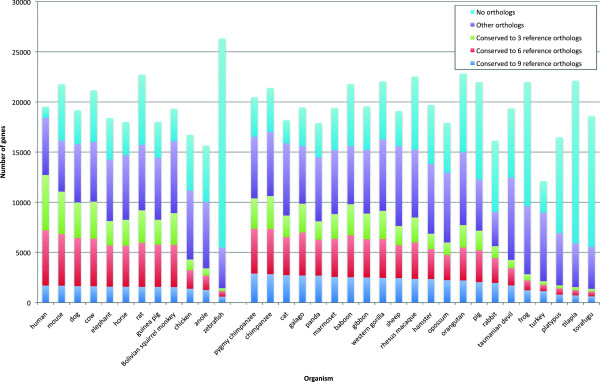
**Extent of conservation in coding regions compared to 12 reference organisms.** To illustrate the degree of splicing conservation between coding regions in each organism and the 12 reference organisms, the most highly conserved genes are identified by conservation to 9 or more reference organisms. Additional categories in decreasing level of conservation are splicing conservation to 6 or more reference organisms, 3 or more reference organisms, and membership in an ortholog set. Reference species are listed before non-reference species.

We assessed the extent of conserved CDS over all 34 vertebrate species. Each protein (in the whole dataset) was compared to its orthologs from the 12 reference species. Only reference species were used in order to reduce the impact of erroneous annotations in lower-quality genomes. These reference species were chosen so that all vertebrate assemblies in scope of RefSeq (and in our dataset) may be evaluated against some close neighbors. Figure 4 provides a snapshot of cross-species support for the CDS in each organism by showing the number of genes with conserved CDS to 3, 6, or 9 reference species. For context, Figure 4 also shows the total number of protein-coding genes for each organism and the number of genes present in ortholog sets. Later, we will discuss using level of conserved CDS to estimate assembly quality.

The number of genes with conserved splicing orthologs appears to be fairly stable across mammals when requiring a splice match to at least 3, 6, or 9 orthologs from reference species; however, the choice of threshold greatly impacts the number of genes labeled as conserved CDS. The median number of genes (across all species) with splice orthologs from 9 out of 12 reference taxa is 1707 compared to 8197 at the 3-reference threshold. Note that reference species exhibit fewer conserved genes at the 6- and 9-ortholog thresholds because each reference gene may be compared to 11 reference species, while all non-reference species were compared to 12 reference species. However, this bias against reference organisms has little impact at the 3-reference threshold. Accordingly, we use the 3-reference threshold as a more inclusive approach to measure the number of genes supported by conserved splicing.

Looking at individual organisms, for human, 70% of genes have evidence of conserved splicing to at least 3 other reference species, a fraction comparable to the human-mouse conservation rate from the previous section. Among all the organisms in our evaluation set, human, mouse, and chimpanzee have the highest splice conservation rates and numbers of conserved genes, possibly reflecting higher annotation quality for human and mouse which have undergone extensive curation efforts. The similarity between chimpanzee and human is expected to have improved annotation of chimpanzee in the NCBI eukaryotic annotation process. Overall, 20 species have over 50% of the genes in ortholog sets with conserved splicing with respect to 3 reference species. Considering the large number of conserved genes and the diversity among its orthologous proteins in both sequence and splicing conservation, we expect that providing information on the scope of conserved splicing will be an interesting addition to RefSeq records.

**Figure 5:**
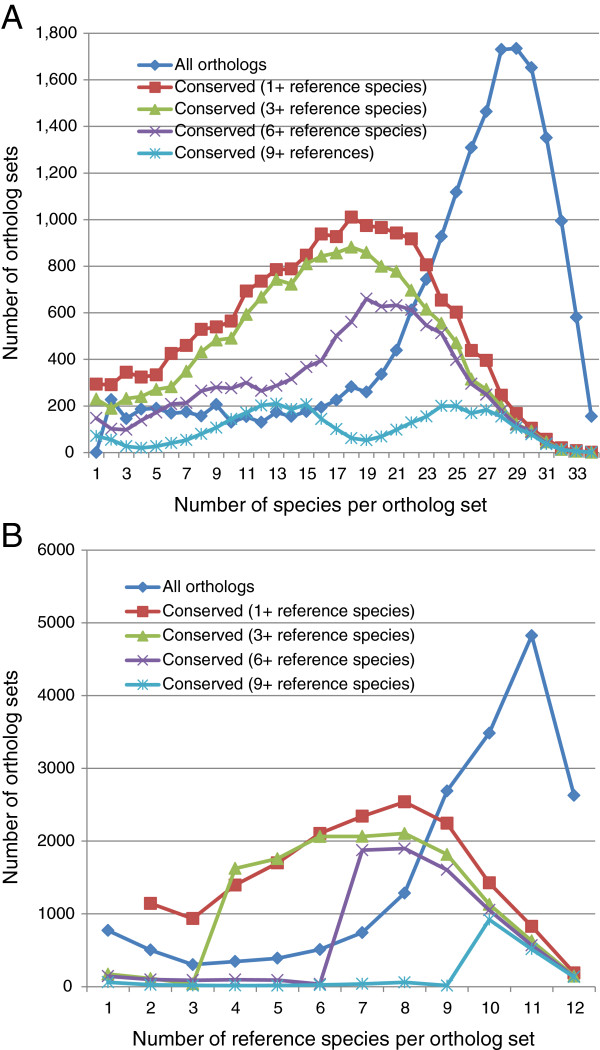
**Distribution of the number of ortholog sets for two categories: total species in ortholog sets (A) where orthologs from non-reference species were compared to the reference species dataset, or (B) restricted to only the reference species in ortholog sets.** Both graphs illustrate the number of orthologs in each category unrestricted by conserved splicing (‘All orthologs’), or as constrained by conserved splicing with increasingly more stringent numbers of reference species. The value of the x-axis is the total number of species in each ortholog set only for the line called “all orthologs”. For all the other lines, the x-axis refers to the number of species in the ortholog set that have splicing conserved at the respective level.

To determine whether a gene is expected to be present in new genomes, we also consider the number of species with conserved splicing within each group of orthologous genes. Figure 5 plots the number of ortholog sets (among all organisms, and across reference organisms only) over two parameters: definition of conserved splicing for each protein (in terms of the number of reference proteins) and number of conserved-splicing proteins in each ortholog set after independently comparing each non-reference ortholog to the orthologs from reference species. The area under the curve ranges from 98% of ortholog sets for 1+ splice orthologs to only 19% of ortholog sets for 9+ splice orthologs. We identify 7,577 genes (or ortholog sets) that are present in at least 17 organisms (half of our evaluation set) with those genes having conserved splicing to at least 3 reference proteins. This forms a potential set of “core proteins” across mammals. Looking at only reference species (Figure 5B), interestingly, there is a peak at 7–9 organisms and a marked drop-off above that, suggesting that previously calculated rates of human-mouse splice conservation can be extended across all mammals with little drop-off. Examples of human genes with splice orthologs in different taxonomic subgroups are listed in Table 5.

**Table 5:** Examples of human genes with splice orthologs across taxonomic subgroups

**Gene ID**	**Symbol**	**Distribution of reference proteins among taxonomic subgroups**				**Sets with conserved splicing**
13	*AADAC*	Primate	Rodent	Mammal	Vertebrate	8584
9	*NAT1*	Primate	Rodent	Mammal		6536
161	*AP2A2*	Primate	Rodent		Vertebrate	100
10	*NAT2*	Primate	Rodent			475
279	*AMY2A*	Primate		Mammal	Vertebrate	167
1	*A1BG*	Primate		Mammal		915
248	*ALPI*	Primate			Vertebrate	21
28	*ABO*	Primate				1233
5005	*ORM2*		Rodent	Mammal	Vertebrate	7
3064	*HTT*		Rodent	Mammal		23
1636	*ACE*		Rodent		Vertebrate	3
1834	*DSPP*		Rodent			29
23284	*LPHN3*			Mammal		29
374	*AREG*				Vertebrate	5

For comparison, the number of ortholog sets containing any genes with conserved splicing orthologs is provided as well.

Please note that splicing conservation of the CDS is determined in pairwise fashion, includes 90% length criteria, and is not necessarily transitive. If gene A has a coding region 90% the length of the corresponding coding region in gene B, and gene B likewise has length 90% compared to gene C, then genes A-B, and B-C are conserved, but genes A and C don’t meet the 90% length criteria and do not have conserved splicing, by our definition. This explains how in Figure 5B, the number of ortholog sets with 1 to 8 genes that are conserved to 9 reference genes is slightly higher than zero, and likewise for the other conservation thresholds in both Figures 5A and 5B.

#### Example: *A2M* alpha-2-macroglobulin

Figure 2 illustrates 10 orthologous alpha-2 macroglobulin proteins from human (GeneID 2) and its orthologs. This gene has a relatively small set of orthologs each encoding a single, richly annotated protein product. Although this orthology set contains similar proteins, the degree of conservation differs when terminal sequence regions and splicing conservation are assessed. Thus, among the reference taxa included in this set of orthologs only human and dog are splicing orthologs. The computationally predicted turkey protein is N-terminally truncated due to a gap in the turkey assembly. The N-terminus of the computationally predicted cat and opossum proteins exhibit greater divergence in length and sequence similarity, respectively. Genome annotation for both is primarily based on protein alignments coupled with *ab initio*, as there is minimal same-species transcript data available. In contrast, the computationally predicted galago protein is of high quality having a conserved N-terminal sequence as well as conserved splicing with 90% overlap in all protein-coding exons compared to human, dog, and guinea pig (all reference taxa); however, human and guinea pig are not splicing counterparts due to length variation in exon 18 (115 bp and 133 bp, respectively). By defining core proteins using a low threshold for the number of reference proteins with conserved CDS, we are able to identify sets of proteins with conserved CDS to at least a few other orthologs, typically from the closest species, without requiring such high level of conservation over all pairs of proteins.

### Cross-species tests for protein consistency

A second application of this protocol is to identify unusual, and potentially mis-annotated, proteins. The two features discussed so far, CDS and sequence similarity, vary widely across different homologs and do not, on their own, indicate problems with the genomic assembly or gene annotations. Instead, we extend our evaluation framework to perform a number of targeted analyses of protein lengths, N-terminal features, and domain composition for protein clusters containing at least 5 reference proteins. Proteins from all 34 species are evaluated using comparisons to the 205,670 proteins from the 12 reference species. Table 6 summarizes the number of proteins that are identified by these tests. A total of 97,367 proteins from 96,635 genes (23% of genes and 17% of proteins in our orthologs dataset) are identified by at least one test and also lack CDS splice conservation (defined as splicing orthologs to at least one protein from the reference species subset). This includes 2.5% of human and 4.6% of mouse genes in our orthology dataset. Some of these discrepancies may reflect real biological differences including annotation differences at the level of alternative splicing, but some of these differences are genuine errors which need to be addressed though improved curation protocols and computational pipeline methods.

**Table 6:** Frequency of outlier proteins by category

**Prefix**	**Jaccard score**	**Extra domain**	**Missing domain**	**Truncated domain**	**Downstream Methionine**	**Length variation**	**N-terminus identity**
NP	572	881	6009	1484	3990	7906	8
XP	3493	7515	61352	13362	26963	93144	1259

XP accession prefix denotes computationally predicted proteins and NP accession prefix denotes proteins that are available for manual curation. These represent targets for evaluation rather than confirmed errors.

#### Domain composition

To measure variation in domain composition, one protein with maximum similarity to orthologs is selected for each gene, that is, the protein with maximum average Jaccard score of domain content [20,21]. A score of 1 indicates that two proteins compared have the same domain composition while a score of 0 indicates no domain in common. Across all species in this study, the average domain score (when calculated) falls within a narrow range of 0.79-0.82. These values are significantly lower than found by [20] which may be due to differences in ortholog identification, domain assignment, and calculation of domain score over only sizable sets of orthologs. Over all genes, 51% had score 1, 13% had score 0, and 6.6% had no score calculated. Using average domain scores for reference species proteins as a sample distribution, a Z-score is calculated for each protein. There are 4065 proteins of interest with a Z-score greater than +/− 2 yields. We also search directly for proteins with extra, missing, or truncated domains compared to all but one of the reference proteins. Unsurprisingly, missing domains are 8-fold more common than extra domains. Some sequence divergence or even a small mis-annotated region may be sufficient to disrupt alignment between a domain PSSM and the sequence, but the presence of an extra domain may point to genuine domain shuffling or long mis-annotated regions.

#### Protein sequence lengths

We identify unusual protein length over the whole protein and within the N-terminal, C-terminal, and conserved regions. First, N-terminal regions are defined as the first 30 and 100 amino acids in each protein (selected to represent short regions and the upper bound on known lengths of mitochondrial transit peptides). A multiple sequence alignment is calculated for each protein cluster, allowing length differences between each protein and all other aligned proteins to be compared. We also define each region based on indel content in the columns of the MSA. Protein lengths are the most common unusual feature detected (see Table 6). However, this is due to a relaxed definition of length outliers that allow length differences as short as 15 amino acids, in order to provide detailed information for review.

#### N-terminal variations

Finally, we searched for two types of errors in the protein N-terminus. First, we looked for alignment of the initial Methionine (Met) amino acid to a downstream Met in multiple proteins, which may point to the less common initial Met being an incorrect translation start position. Requiring either the upstream or downstream Met to be conserved in at least half of the comparisons to proteins from the reference species proteins dataset returns 30,953 proteins. Our protocol has already clustered alternative splicing products to their closest counterparts however the majority of proteins in the dataset are inferred from predicted gene annotations and for many genes, only one protein product is predicted. Consequently, our results indicate that some of these predicted proteins may have incorrect translation start positions, while other genes may encode additional products with the more conserved translation start [22].

A second type of error at N-terminal involves exons annotated at the incorrect genomic location. N-terminal coding exons are frequently more distant from the remaining coding exons and more challenging to annotate in computational pipelines when there is scant same-species transcript data available to specifically define the exon boundaries and when homologous protein alignments do not extend to the N-terminus due to cross-species sequence differences, masking of genomic sequence, or indels or larger gaps in the assembled genomic sequence. We attempt to identify such errors using sequence similarity: proteins with particularly poor sequence similarity at the N-terminal compared to its orthologs and compared to whole-sequence similarity are candidates for incorrect N-terminal coding exons. Testing on N-terminal regions defined as initial 30-residue or 100-residue regions identifies 1267 proteins that need curator review.

Our results provide a summary of specific, consistent differences in particular proteins, which may be valuable for internal review to improve the manually curated dataset and to identify targets for improvement of NCBI’s genome annotation pipeline.

### Estimating annotation quality

The tests described previously have generated a number of statistics related to conservation or lack of consistency. Here we define a score for annotation quality that is independent of assembly quality measures (specifically the contig N50), or support evidence measures (here approximated by number of same-species ESTs). Our score leverages the preceding methods for sequence conservation and splice orthologs and is based on the above tests for unusual protein sequence properties. For each organism, we count the number of genes with the following properties: 1) outlier domain (Jaccard) score outside average range for analyzed species; 2) extra, missing, or truncated domain; 3) outlier length (as described in Methods); 4) conserved downstream Met aligned to initial Met (or vice versa); and 5) absence of protein in the largest protein cluster (although present in other clusters). An aggregate score is calculated as the negative log of the product of all scores (see Table 7). This combined score is a variation of weighted average scores that applies a log transformation to each score to help to equalize contributions from all methods instead of favoring those with larger scores. Since the true annotation or assembly quality is not known, we use contig N50s to approximate annotation quality as availability of transcript data and assembly quality are both known to influence the outcome. The contig N50 is a statistical measure such that 50% of the bases in the genome assembly are found in the subset of contigs of this length or longer. It is commonly used as a simple metric of assembly quality where a higher contig N50 value is an indicator of a higher quality assembly.

**Table 7:** Estimating annotation quality using protein features and sequence data

**Organism**	**Domain score outlier**	**+,-,truncated domain**	**Length outlier**	**Conserved downstream Methionine**	**No protein in main cluster**	**Negative sum-of-logs score**	**Core proteins**
*human	0.000	0.021	0.017	0.028	0.012	10.256	7419
*housemouse	0.002	0.033	0.036	0.051	0.049	8.135	6811
*dog	0.005	0.068	0.115	0.051	0.030	7.268	6495
*African savanna elephant	0.006	0.096	0.163	0.038	0.042	6.816	5685
*domestic guinea pig	0.005	0.093	0.158	0.047	0.045	6.812	5699
*cow	0.008	0.082	0.101	0.041	0.060	6.795	6394
pygmy chimpanzee	0.003	0.100	0.146	0.081	0.044	6.764	6584
*horse	0.006	0.105	0.149	0.054	0.042	6.699	5838
rabbit	0.004	0.122	0.189	0.050	0.052	6.646	4159
chimpanzee	0.005	0.108	0.138	0.068	0.048	6.600	6455
*norway rat	0.008	0.077	0.084	0.061	0.080	6.600	5851
giant panda	0.005	0.139	0.224	0.063	0.035	6.501	5765
olive baboon	0.006	0.115	0.156	0.077	0.048	6.416	5872
*bolivian squirrel monkey	0.007	0.108	0.152	0.086	0.053	6.284	6035
white-tufted-ear marmoset	0.005	0.126	0.207	0.083	0.055	6.197	5777
domestic cat	0.005	0.193	0.254	0.070	0.041	6.178	6007
northern white-cheeked gibbon	0.006	0.148	0.195	0.077	0.056	6.151	5730
western gorilla	0.007	0.167	0.197	0.078	0.057	5.958	5722
small-eared galago	0.012	0.138	0.197	0.076	0.046	5.955	6128
gray short-tailed opossum	0.006	0.214	0.370	0.058	0.041	5.924	4500
chinese hamster	0.006	0.190	0.265	0.066	0.061	5.892	4901
sheep	0.007	0.232	0.292	0.070	0.041	5.871	5339
*chicken	0.013	0.175	0.228	0.072	0.051	5.729	3354
rhesus macaque	0.011	0.169	0.216	0.083	0.072	5.639	5466
pig	0.025	0.192	0.230	0.061	0.042	5.551	4616
sumatran orangutan	0.014	0.169	0.222	0.081	0.071	5.509	5050
*green anole	0.018	0.215	0.296	0.068	0.048	5.431	2797
nile tilapia	0.011	0.392	0.583	0.071	0.061	4.961	1296
tasmanian devil	0.016	0.337	0.478	0.082	0.054	4.933	3457
*zebrafish	0.025	0.344	0.464	0.062	0.068	4.773	1210
torafugu	0.017	0.448	0.601	0.060	0.063	4.765	1130
turkey	0.023	0.425	0.520	0.077	0.055	4.659	1821
western clawed frog	0.023	0.394	0.484	0.073	0.070	4.644	2268
platypus	0.031	0.435	0.586	0.068	0.067	4.451	1439
Average	0.010	0.187	0.256	0.066	0.052	6.111	4796
Standard deviation	0.008	0.120	0.160	0.014	0.014	1.114	1826
Correlation Contig N50	−0.290	−0.337	−0.362	−0.489	−0.434	0.719	0.319
Correlation EST count	−0.164	−0.299	−0.342	−0.555	−0.395	0.684	0.263

Columns 2–7 provide fraction of genes from each species in the orthology dataset and lacking conserved protein; outlier domain score; missing, extra, or truncated domain; outlier length; conserved downstream or upstream Met; and proteins not found in largest cluster. Column 8 provides the negative sum of logs score. The last column provides the number of genes in each species that are members of a “core” set defined as ortholog sets with members from at least 17 species that each have conserved splicing to at least 3 proteins from reference species (reference species are marked with *.)

To estimate whether these criteria can be used to help gauge annotation quality, we calculate the Pearson correlation coefficient for each method. The 6 individual methods have an inverse correlation with contig N50 ranging from −0.29 to −0.49 while the sum-of-logs score has higher correlation 0.72 (p-value < 0.00001). (The respective values calculated using EST counts are very similar.) Interestingly, among the individual methods, the test of conserved upstream/downstream Met has strongest correlation compared to all other criteria, and its Spearman correlation coefficient is even stronger (−0.7). This likely indicates a deficiency in the NCBI eukaryotic annotation pipeline specific to correctly annotating N-terminal regions. Correlations are higher when calculated separately for each taxonomic subgroup Excluding human, mouse, and zebrafish which have outlier contig N50 values, the Pearson correlation coefficient was 0.88 for primates, 0.95 for rodents, 0.67 for mammals, and 0.87 for vertebrates (all p-values < 0.03). These results confirm that the combined “error” score may be valuable for estimating quality, especially by comparing scores between close species.

We note that the number of core proteins in each species is only weakly correlated with contig N50s (correlation 0.32) and did not boost performance of the combined score with respect to a stronger correlation coefficient. Here, core proteins were defined as the splice-conserved proteins in the 7,577 gene (ortholog) sets that each contain at least 17 proteins having conserved splicing to 3 or more reference proteins (as described in a previous section). Nevertheless, we may use the number of core proteins to supplement the combined score as the former is independent to the combined score and more easily interpreted, as a direct measurement of the extent of gene conservation. We note that in contrast the total number of protein-coding genes in each species bears no correlation with contig N50; this could imply that a sizable number of protein-coding genes are species specific or erroneous.

Within the primate subgroup, the correlation between the number of core proteins and contig N50s for all species besides human was particularly strong at 0.82 (p-value < 0.003). Indeed, both the combined score and the number of core genes are low for macaque which is known to have a poorer quality genome assembly [23], and likewise predicted (based on a lower contig N50) for orangutan. In contrast, gorilla, which has a new assembly based on Sanger and Solexa sequencing, has a larger number of core proteins and higher combined score despite a lower contig N50. This indicates that our approach is a more sensitive metric for annotation quality than N50 or EST count alone. For mammals, platypus, Tasmanian devil, and pig have low combined score while cat and panda have higher scores despite scarcity of contig N50s or ESTs. These results exemplify how directly evaluating conservation across orthologous genes provide more sensitive measures of overall annotation quality.

## Conclusions

RefSeq is in a unique position to provide orthology and comparative analysis results, as a repository of genome-wide high-quality gene, transcript, and protein records, and having access to resources hosted by NCBI and other sites. An efficient hybrid method for orthology identification has recently been put into production to provide expanded quality assurance for curated RefSeq proteins and identify areas to target improvements in the genome annotation pipeline. These results supplement the data available in HomoloGene. Taking advantage of the extensive orthology data available, we have developed a computational pipeline to perform several orthogonal analyses on these orthology sets. The process described here has been incorporated into regular RefSeq processing: it is run regularly in response to newly annotated genome assemblies, changes in the gene membership of ortholog sets, and changes (updates and additions) to the protein products of each gene. Employing parallel processing resources enables results for the 568,459 proteins in our dataset to be calculated within hours, and this process can be adapted to scale linearly to accommodate growth in the number of genomes.

Using our suite of methods, we demonstrated a high level of consistency in RefSeq protein representation among vertebrates. Independent assessment measures that include considerations of protein sequence similarity, exon coverage, and splice similarity provide similar results. Previous comparisons of human and mouse orthologs have reported identical splicing in 32% of transcript pairs, and 64% highly conserved splice orthologs with a relaxed criteria that tolerates exon length differences of up to 5 codons [9], and identical lengths in 73% of corresponding human-mouse exons within a smaller data set [10]. Our results of 71% splice orthologs between human and mouse and 68% splice orthologs between human and cow are consistent with these previous reports but we offer a considerably expanded dataset scope. These results lend support to conclusions of the quality of RefSeq proteins for organisms beyond human and mouse and provide specific information about the most conserved protein annotations. These results suggest that within a relatively narrow taxonomic scope such as mammals, many orthologs can be expected to have similar structure in their protein-coding exons, and that comparison of splicing is a reasonable metric for distinguishing counterparts among the various isoforms in orthologous genes.

We find that the majority of the RefSeq vertebrate proteins for which we have calculated orthology are good as measured by several orthogonal metrics (number of orthologs in sets, splice conservation, protein tests), and we find particular concern in N-terminal sequence definitions. Furthermore, our results suggest that evaluating annotation results for unusual sequence qualities is a reasonable metric for annotation quality that is independent of available transcript data and contig N50. Our findings agree with previous reports of lower quality annotation for rhesus and our aggregate error score may be a generally useful measure of overall annotation quality for a given genome (a more direct and granular metric than contig N50 although there is a correlation with contig N50).

Novel genomes of interest may contain few highly-conserved genes compared to the organisms in our evaluation set, particularly organisms have been shown to be genomic “singletons” with few close relatives [24]. We have attempted to assuage this issue by selecting a representative set of organisms related to the most commonly analyzed mammals. We also showed here that thousands of genes in mammals that are relatively distant from primates and rodents are highly conserved compared to 3 or more reference species. Consequently we expect to be able to reuse this reference set of genomes to evaluate a sizable fraction of genes in a variety of mammals. Further, our computational pipeline may be applied to a different set of organisms. For a finer-level evaluation of novel genomes, we can further refine our process flow to identify genome neighbors [24] and apply the process described here using that customized set of species for comparison. Note that our method relies on coverage of proteins from those organisms in Swiss-Prot and availability of accurate assembly data, so such an approach would still have some shortcomings.

The process flow described here is being incorporated into the suite of RefSeq analysis protocols and results will be used multiple ways including: a) identify outliers needing the attention of RefSeq curation staff; b) provide additional public information about proteins with higher conservation as well as protein isoforms that are predicted to be more functionally relevant (or of uncertain function) based on the annotation signatures of signal peptide and domain content; c) as a quality assurance benchmark for annotation of new vertebrate (especially mammalian) genomes in that the most conserved protein dataset should reasonably be expected to be annotated; and d) to further improve the NCBI eukaryotic genome annotation pipeline.

## Methods

### Dataset of orthologous genes

Orthologous genes in human and 33 additional vertebrates (Table 2) were identified using a hybrid method of protein homology and local synteny. RefSeq proteins and Gnomon models for each taxa were queried against the Swiss-Prot database [3] using BLASTp with default parameters, and the best hit selected based on bit score with at least 50% query coverage. Proteins with best hit to Swiss-Prot proteins with the same name (for example “Alpha-2-macroglobulin”) are considered potential homologs to one another. At present all sets are “anchored” around a human gene: each set of homonymous proteins must contain a human protein with respectively named counterpart in Swiss-Prot.

Their respective genes are confirmed as orthologs if at least two of the six flanking genes (three on each side) for each gene are in the expected order and orientation or if the genes are single copy in both the human and the target species and share at least one of the six flanking genes. For this study, only genes that can be mapped to a reference genome assembly are retained, to ensure that exon annotations can be calculated against the same reference assembly. For simplicity, only non-redundant proteins are retained for this evaluation. One gene can encode multiple, identical proteins in the database when they correspond to different transcripts with the same coding regions.

### Clustering splice variants

Genes in this dataset, primarily from human and mouse, may encode one or more proteins (due to alternate splicing). Protein sequence similarity was used to cluster proteins in each ortholog set to identify the most similar proteins among alternative splicing variants. Within each set of orthologous genes, pairwise alignments were calculated between all proteins from reference species and all proteins in the set using BLAST [25] with parameters E-value 1e-05, no composition-adjusted statistics, and no masking, and BLAST scores were used to determine reciprocal best hit proteins for each pair of genes (species) with alignments. Ties are broken by selecting the protein with length closer to the query protein. Among identical proteins for one gene, as may occur when the gene has UTR-specific splice variants, one protein is randomly selected to be the representative protein. Proteins are then clustered into sets of “orthologous proteins” such that no set contains more than one protein from each gene, according to a simple greedy algorithm: Analyzing reciprocal best hits in order of descending BLAST score, the two proteins are merged into the same cluster (or rather, their respective clusters are merged) if this operation does not create a cluster containing two proteins from the same gene.

### Detecting conserved coding exons

Exon junctions for each transcript are extracted from pre-existing mRNA feature annotations on the RefSeq record when they exist, or computed through cDNA-genome alignments using Splign [17]. Genomic locations for each gene are obtained from database for the current reference assembly. The pairwise protein-protein alignments described in the previous section define the corresponding nucleotide positions between their respective mRNAs as well. Exon boundaries are then mapped onto the transcript sequences providing inferred alignments between protein-coding exons, enabling us to test conservation across exon positions and splice junctions. A few adjustments are made to reduce the impact of incomplete protein and mRNA-genome alignments: The alignments between the exons at the N- and C-terminals of the protein alignment are extended gap-free to the beginning and end of the respective exons. When a transcript cannot be wholly aligned to the selected genomic region, nucleotide positions outside of a defined exon are labeled as part of the previous exon, except for positions preceding the first exon.

### Protein functional annotations

For each proteins, domain from the Conserved Domain Database [26] are assigned from protein sequence using the CD-Search tool [27]. These values are pre-calculated and contained in the protein record viewable in NCBI’s Protein resource [15]. We use all domains in CDD v3.08 that have been clustered into superfamilies, which include all NCBI-curated and imported models except certain models that span multiple other domains. Additionally, signal peptides are predicted for all sequences using SignalP4.0 [28].

For proteins in clusters containing at least 5 proteins from reference species, we calculate unusual domain content at the level of CDD superfamily annotations (plus signal peptides). First, domain similarity is calculated between all proteins against reference proteins using Jaccard index. The Jaccard score for domains in proteins P and Q is defined as

$$\begin{array}{l} \mathrm{JC}\_\mathrm{score}\left(P,Q\right)=\mathrm{P\cap Q}/\mathrm{P\cup Q}\\ \;=\left|\mathrm{domains}\;\mathrm{in}\;P\&Q\right|/\left|\mathrm{domains}\;\mathrm{in}\;P\right|\\ \;+\left|\mathrm{domains}\;\mathrm{in}\;Q\right|–\left|\mathrm{domains}\;\mathrm{in}\;P\&Q\right|\; \end{array}$$

Z-scores for different scores, including Jaccard indices, were calculated using reference proteins to determine mean and standard deviation.

Second, proteins with extra, missing, and truncated domains compared to at least *r-1* reference proteins (where *r* is the number of reference proteins) are flagged. We use *r-1* to enable testing of the reference proteins themselves. Since CDD superfamily annotations are based on alignments between the protein and a CDD domain in the superfamily cluster, we define domain truncation as the maximum hit length fraction (length of alignment between PSSM and protein sequence, divided by PSSM length) is less than 60% of the PSSM length over one or more occurrences of that superfamily on the protein; this maximum hit length fraction is also required to be less than 60% of the hit length fraction for this superfamily in all other proteins in its cluster.

### Identifying sequence and length outliers

For clusters of proteins with at least 5 members from reference species, multiple sequence alignments were calculated using COBALT [29]. Then, we performed a suite of calculations for length, N-terminal, and sequence outliers for each protein compared to reference proteins:

1) Average ungapped sequence identity over the first 30 and 100 residues, flagging proteins with identity < 50%, Z-score of identity greater than +/−2. To aid in screening out cases with low sequence identity over the whole sequence, we also require the Z-score of log( identity over N-terminal region / identity over whole sequence) to be greater than +/− 2.

2) Average length difference at N-terminal compared to reference protein, flagging proteins with length difference greater than 15 and Z-score of length difference greater than +/− 3.

3) Identify proteins with initial methionine codon aligned to a downstream methionine in another protein, or vice versa, where this difference is preserved across more than (*r-1)/2* proteins.

To increase sensitivity in detecting length outliers that might be masked by similar whole-protein length, we also label regions of each multiple sequence alignment as conserved, N-terminal, C-terminal, or intermediate regions by requiring at most 20% gap content across reference proteins within conserved regions. Trailing terminal sequence regions not present in the MSA are included in the calculations. Then, we report proteins with length difference across any region or whole protein with Z-score greater than +/− 3 or absolute length difference greater than 15 compared to *r-1* reference proteins.

### Data access

The RefSeq proteins used in this analysis are publicly available at NCBI [30]. A supplemental data file listing the protein identifiers and related information is provided on the RefSeq ftp site [31].

## Abbreviations

BLAST: Basic local alignment search tool; Bp: Base pair; CDD: Conserved domain database; CCDS: Consensus coding sequence; CDS: Coding sequence; COBALT: Constraint-based multiple protein alignment tool; Indel: Insertion or deletion; NCBI: National Center for Biotechnology Information; PSSM: Position specific scoring matrix; RefSeq: Reference sequences; Tax ID: NCBI taxonomy identifier; UTR: Untranslated region.

## Competing interests

The authors declare that they have no competing interests.

## Authors’ contributions

JF carried out analysis of the data and wrote the manuscript. TM calculated the ortholog dataset, provided technical support for database storage, and wrote sections of the manuscript. KP conceived of the study, participated in design and coordination, and helped to draft the manuscript. All authors read and approved the final manuscript.

## Authors’ information

JF worked at the National Institutes of Health during the period of time that this work was carried out and the manuscript was written. She was not affiliated with the NIH at the time of manuscript submission.
